# Model-Independent Lens Distortion Correction Based on Sub-Pixel Phase Encoding

**DOI:** 10.3390/s21227465

**Published:** 2021-11-10

**Authors:** Pengbo Xiong, Shaokai Wang, Weibo Wang, Qixin Ye, Shujiao Ye

**Affiliations:** 1 Institute of Ultra-Precision Optoelectronic Instrument Engineering, Harbin Institute of Technology, Harbin 150001, China; 19b901031@stu.hit.edu.cn (P.X.); wangsk@hit.edu.cn (S.W.); 19s001037@stu.hit.edu.cn (Q.Y.); yeshujiao@stu.hit.edu.cn (S.Y.); 2 Key Lab of Ultra-Precision Intelligent Instrumentation, Harbin Institute of Technology, Ministry of Industry and Information Technology, Harbin 150001, China; 3 Postdoctoral Research Station of Optical Engineering, Harbin Institute of Technology, Harbin 150001, China

**Keywords:** camera calibration, fringe pattern, phase encoding, model-independent method

## Abstract

Lens distortion can introduce deviations in visual measurement and positioning. The distortion can be minimized by optimizing the lens and selecting high-quality optical glass, but it cannot be completely eliminated. Most existing correction methods are based on accurate distortion models and stable image characteristics. However, the distortion is usually a mixture of the radial distortion and the tangential distortion of the lens group, which makes it difficult for the mathematical model to accurately fit the non-uniform distortion. This paper proposes a new model-independent lens complex distortion correction method. Taking the horizontal and vertical stripe pattern as the calibration target, the sub-pixel value distribution visualizes the image distortion, and the correction parameters are directly obtained from the pixel distribution. A quantitative evaluation method suitable for model-independent methods is proposed. The method only calculates the error based on the characteristic points of the corrected picture itself. Experiments show that this method can accurately correct distortion with only 8 pictures, with an error of 0.39 pixels, which provides a simple method for complex lens distortion correction.

## 1. Introduction

The optical aberration of the lens will cause the non-linear distortion of the image. Distortion correction is necessary in digital image analysis. For example, computer vision tasks involving geometric location, size measurement, image recognition, and accurate distortion correction are essential [1,2,3,4]. The existing distortion correction methods can be subdivided into three categories: traditional vision calibration, active vision calibration, and learning-based methods [5,6,7].

In traditional vision measurement methods, geometric features such as corner points, vanishing points, and straight lines are the main control targets for correction [8]. The characteristic of the traditional methods is to use the known structure information of the scene, which is usually used to calibrate the block. It can be used for any camera model with high calibration accuracy. However, the calibration process is complicated and requires high-precision known structural information. In many cases, the calibration block cannot be used in actual applications. The accuracy depends on the density of geometric feature selection and is invalid for nonlinear distortion. Therefore, Zhang [9,10] proposed a multi-viewpoint correction method that uses the correspondence between points in different images to measure lens distortion parameters. It does not require special operations to realize automatic correction, but a set of images to establish a mathematical model. The method and its improved version are more flexible calibration methods, which can calculate the internal and external parameters of the camera by capturing 3 to 20 patterns at different positions and angles, thereby achieving high-precision camera calibration. However, in some special cases, non-radial, tangential nonlinearity, or hybrid distortion may not be well corrected because the distortion is the cumulative effect of complex lens systems, camera geometric errors, and sensor shapes. The distortion mathematical model of the traditional method cannot fit the actual distortion well. An overly accurate model is bound to be very complicated, and the coupling of parameters may also lead to numerical instability.

The calibration method based on radial alignment constraint (RAC), proposed by Tsai, is an important task of computer vision camera calibration. The core of this method is to use the radial uniform constraint to solve other camera external parameters except for the translation of the camera’s optical axis direction, and then solve other camera parameters. The biggest advantage of the RAC-based method is that most of the equations it uses are linear equations, which reduces the complexity of parameter solving, so its calibration process is fast and accurate. However, this method only considers radial distortion. 

In recent years, the camera calibration based on the circle calibration method is similar to Zhang’s. The template image is taken from at least three different directions, and the coordinates of the ring point image on each image are calculated according to the projection invariance to obtain the internal parameter matrix. At least six equations can solve all internal parameters. The method is based on curve fitting (stable) and does not require any matching, while Zhang’s method is based on points and needs to match template points and image points. In addition, parallel circle calibration method only needs to start from the fitted quadratic curve, does not need any matching, does not need to calculate the center of the circle, and has a wide range of applications.

The active visual calibration methods are mainly based on the orthogonal motion method of the plane homography matrix and the orthogonal motion method based on the outer pole. Compared with the most classic active vision calibration method, these methods have the following advantages: the camera’s two-orthogonal motion is easier to implement than the three-orthogonal motion, all five internal parameters of the camera can be solved. 

In the learning-based method, the data set still prioritizes the image set generated by the specific distortion mathematical model [11,12,13,14,15]. However, any existing mathematical distortion model cannot be well applied to all actual shots with manufacturing artifacts, and the training set cannot be applied to any target.

In order to solve the problems of traditional methods and learning-based methods, scholars have proposed non-model distortion correction methods. Thirthala [16] proposed a method to recover radial distortion using multi-focus tensor, but this method is based on the assumption that the distortion center is located in the center of the image. Xu Y. [17] proposed a camera calibration method based on an iterative distortion compensation algorithm; the initial parameters of the camera are calibrated by the pixels of the full-field camera and the corresponding points on the phase target. Although this method can achieve higher accuracy, it is still based on the calculation of internal and external parameters of the initial parameters, and pixel-by-pixel compensation. It may not be suitable for lens groups or complex imaging systems. Based on the above analysis, the traditional modeling and learning-based correction methods have some common problems to be solved. Model-independent methods are a new trend in industrial lens distortion correction.

The phase analysis of structured light is widely used in various optical measurement techniques such as interferometry and digital holography, due to its high accuracy [18,19]. It has also been introduced into the method of lens distortion correction. For example, Liu Dan [20] proposed a calibration method for lens distortion correction of cameras using vanishing points and ellipses in a single image. Yoneyama [21,22] used digital image correlation and speckle pattern or cross grating pattern as a reference to study distortion correction for accurate displacement measurement. However, for such methods, the accuracy depends on the density of feature points. We are looking for a method with high accuracy, simple operation, simple principle, and capable of coping with complex distortion.

In this article, we propose a model-independent lens distortion correction method based on sub-pixel phase encoding and isophase detection. Unlike other methods, our purpose is to obtain the pixel distribution parameters of the entire field of view. In this method, the camera’s field of view (FOV) is located inside the liquid crystal display (LCD), and horizontal and vertical sinusoidal grating fringe images are captured, and the pixel coordinates are stored as correction parameters. First, the camera pixels are sub-pixel encoded using horizontal and vertical grating fringe patterns and a four-step phase shift algorithm. Then, the correction parameters are obtained through the isophase coordinate detection method. Distortion correction is sub-pixel resampling of the captured image. This method is completely based on the actual distortion produced by the lens and camera combination. Radial distortion, caused by optical aberrations, and tangential distortion, caused by lens and camera installation, are corrected at the same time. Finally, a quantitative evaluation method for distortion correction based on checkerboard angles is proposed. By comparing the quantitative results, the effect can be obtained intuitively.

## 2. Methods

### 2.1. Basic Model of Lens Distortion

**Figure 1:**
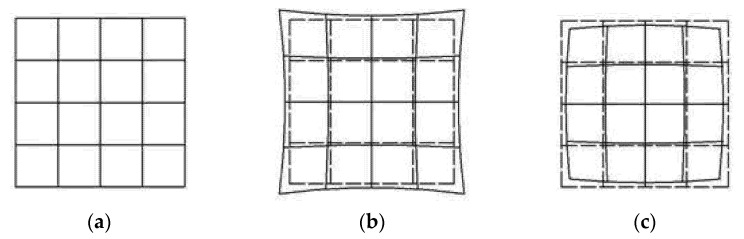
(**a**) No distortion; (**b**) pincushion distortion; (**c**) barrel distortion.

Lens distortion is caused by difference of magnification, which makes the light bend more in the area far from the center of the lens. The distortion at the center of the optical axis is 0, and increases moving along the radius of the lens to the edge. Two kinds of radial distortion are shown in Figure 1. The mathematical model of distortion can be described by the first few terms of the Taylor series around the principal point. Generally, the distortion is described by the first two or three terms (*k*_1_, *k*_2_, and *k*_3_), and the formula is:

(1) $$x_{0}=x\left(1+k_{1}r^{2}+k_{2}r^{4}+k_{3}r^{6}\right)$$

(2) $$y_{0}=y\left(1+k_{1}r^{2}+k_{2}r^{4}+k_{3}r^{6}\right)$$

In the formula, (*x*_0_, *y*_0_) is the position where the light is imaged on the camera sensor when there is no distortion, and (*x*, *y*) is the actual position after the distortion occurs. The farther the pixel point is from the optical center, the greater the radial displacement (*r*), the greater the distortion, and there is almost no distortion near the optical center. Note that the above model is only a rough representation of the distortion distribution.

### 2.2. Phase Encoding and Isophase Detection

**Figure 2:**
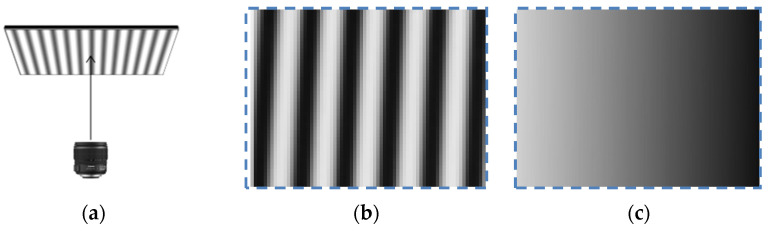
(**a**) The lens is parallel to the LCD; (**b**) captured fringe image; (**c**) sub-pixel encoding.

In order to obtain accurate distortion parameters of pixels at different distances from the optical center, the accuracy needs to reach the sub-pixel size of the camera sensor. The phase shift method is widely used in optical metrology and is suitable for accurate feature extraction. The camera and LCD are placed in parallel. Figure 2 shows the sub-pixel phase encoding of the camera sensor. The sub-pixel encoding is achieved through linear interpolation to obtain a new phase map that is twice the size of the original one.

From the light intensity distribution function, the expression of the four-step phase shift method is as follows:

(3) $$I_{n}\left(x,y\right)=I_{0}\left(x,y\right)+A\left(x,y\right)\cos\left[\varphi \left(x,y\right)+\left(n-1\right)\pi /2\right],n=1,2,3,4$$

$I_{0}\left(x,y\right)$ is the background light intensity, A(x,y) is amplitude, (n−1)π/2 is phase shift, φ(x,y) is unknown dominant phase value, which can be solved by Formula (4):

(4) $$\varphi \left(x,y\right)=\arctan\left(\frac{I_{4}-I_{2}}{I_{1}-I_{3}}\right)$$

**Figure 3:**
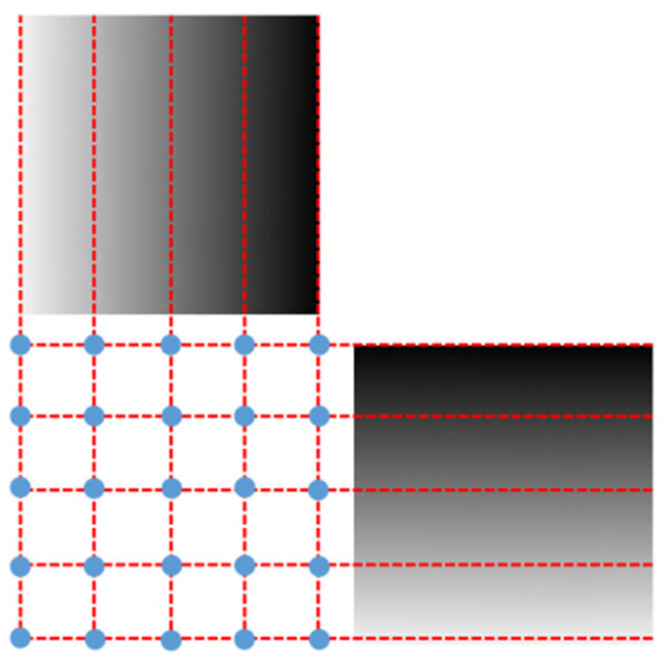
A schematic diagram of a standard pixel array structure.

Take the horizontal phase diagram as an example. Starting from the first pixel, search for pixels in each column that have the same phase value as the starting pixel. If the result is empty, it means that the initial phase value is not continuous in phase. Then, stop searching and skip to the next line and repeat the above steps until a continuous and complete isophase appears. The isophase line at the edge of the image is regarded as the edge isophase line. According to the characteristic that the phase of the grating fringe is uniformly spaced and linearly distributed on the liquid crystal display, a standard pixel array can be constructed through interpolation. Figure 3 is a schematic diagram of a standard pixel array structure. The method of the vertical phase diagram is the same. 

The standard pixel array is composed of *x* rows and y columns (the values of *x* and *y* are twice as the number of camera sensor pixels due to interpolation); the isophase lines obtained from the horizontal phase diagram, the phase values are, respectively, *a*, *b*; the isophase lines obtained from the vertical phase diagram, the phase values are *m* and *n*, respectively. For each standard pixel, it contains two phase encoding values, a horizontal phase encoding value *h* and a vertical phase encoding value *v*. Then, for the standard pixel *P_ij_* (*h, v*) in the *i*-th row and *j*-th column:

(5) $$P_{ij}(h,v)=P_{ij}(a+(i-1)\frac{\left|b-a\right|}{x-1},m+(j-1)\frac{\left|m-n\right|}{y-1}),i\in \left[1,x\right],j\in \left[1,y\right]$$

### 2.3. Obtaining Parameters and Correction

**Figure 4:**
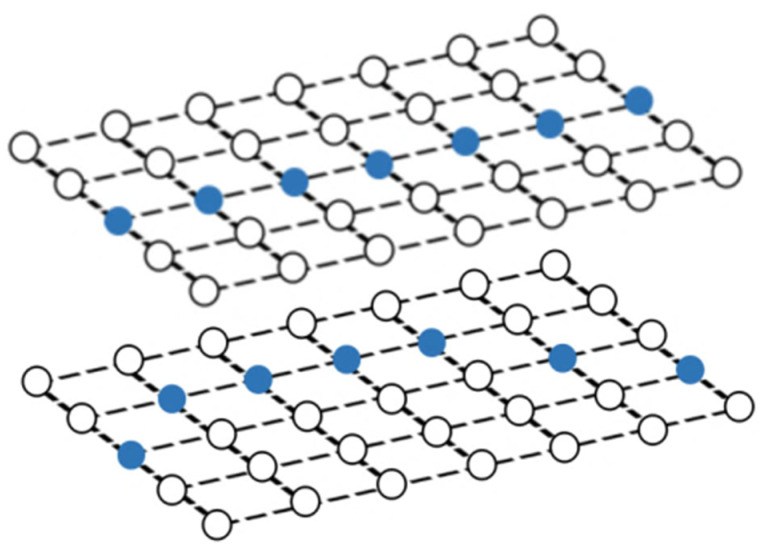
Resampling: assign the gray value of the image to be corrected to the standard pixel array according to the recorded coordinates.

**Figure 5:**
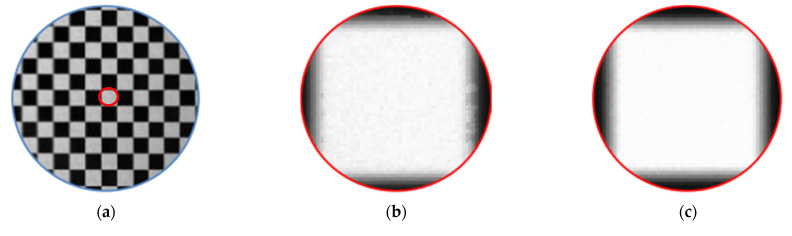
(**a**) Example of corrected checkerboard image; (**b**) the image becomes blurred due to the environment and hardware; (**c**) partial enlargement of the corrected image after parameter optimization.

**Figure 6:**
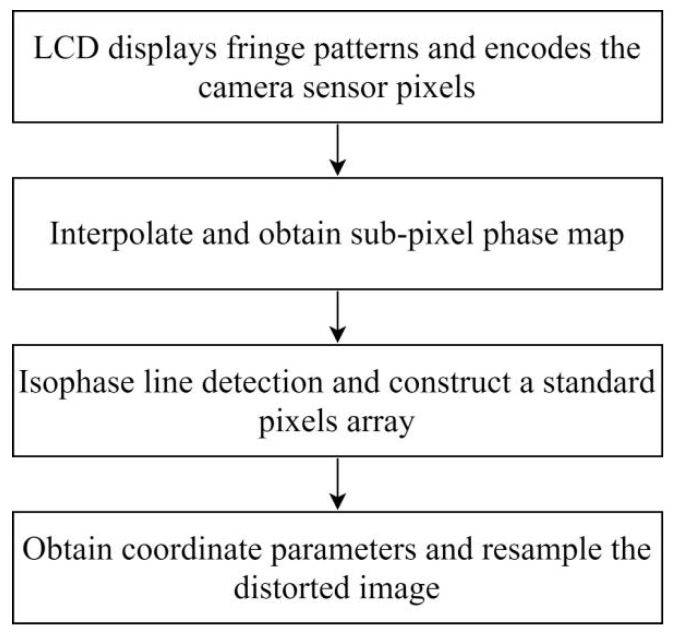
The process of distortion correction.

After constructing the standard pixel array, take the phase of a certain pixel of the standard array as the target value (each pixel contains two phase encoding values), find matching pixels in the sub-pixel phase map of the image, record the pixel coordinates, and use the correction parameters. Resample the linearly interpolated image. Resampling is to assign the gray value of the image to be corrected to the standard pixel array according to the recorded coordinates, as shown in Figure 4. Affected by the hardware and environment, the phase encoding will also deviate, and the resampled image will be blurred, as shown in Figure 5b. Therefore, the correction parameters are smoothly corrected to eliminate impulse points, as shown in Figure 5c. Finally, restore the corrected picture to its original size. The calibration process is shown in Figure 6.

### 2.4. Quantitative Evaluation

**Figure 7:**
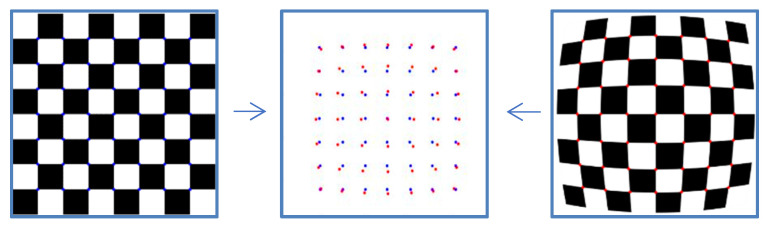
The deviation of the corner points of the distorted checkerboard (red) from the corner points of the standard checkerboard (blue).

**Figure 8:**
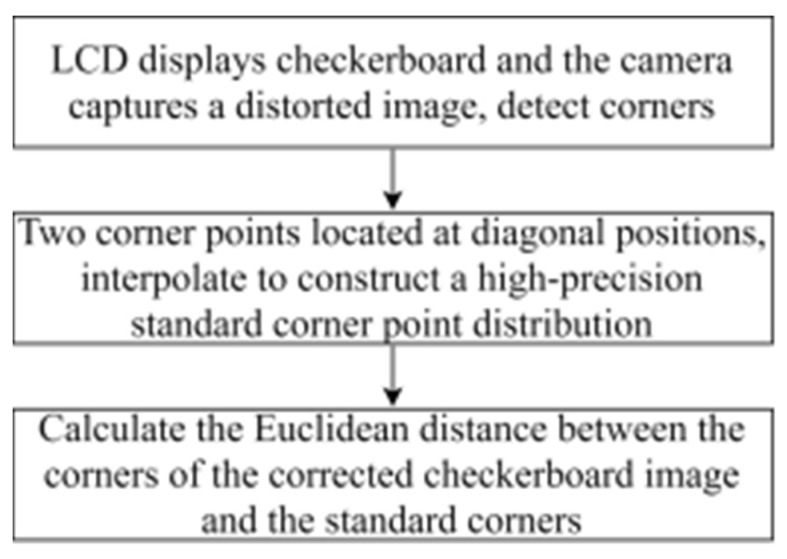
The process of quantitative evaluation.

The corner points of the checkerboard are strictly distributed, which are used to quantify the effect of distortion correction. The effect of distortion correction can be judged only by analyzing the position of the corner points of the corrected checkerboard image. Thus, the quantitative evaluation of distortion correction can be obtained by calculating the Euclidean distance between the corner points of the corrected image and those of the standard checkerboard. The standard distribution of the corner points can be obtained by fitting two corner points of the corrected image with high fitting accuracy. Figure 7 shows the deviation of the corner points of the distorted checkerboard (red) from the corner points of the standard checkerboard (blue). Figure 8 shows the quantitative evaluation of the correction. The coordinate of any corner of the corrected image is (*x_i_*, *y_i_*), and the coordinates of the corners of the standard checkerboard corresponding to that is (*x_i_*’, *y_i_*’). For any corner point, the error is expressed as:

(6) $$e_{i}=\sqrt{{\left(x_{i}-{x_{i}}^{\prime }\right)}^{2}+{\left(y_{i}-{y_{i}}^{\prime }\right)}^{2}}$$

For an image with *n* corner points, the average error can be expressed as the average of the Euclidean distances of corner points:

(7) $$\bar{e}=\frac{\sum_{i=1}^{n}e_{i}}{n}$$

### 2.5. Numerical Simulation

Numerical simulation is performed to verify the validity of the method we introduced. Firstly, two sets of longitudinal and transverse sinusoidal fringe patterns of 512 × 512 pixels are employed as measuring patterns. Then, the essence of distortion simulation is coordinate transformation. The gray value of each pixel coordinate point in the original image is assigned to the corresponding coordinate point obtained by the distortion model, and the gray value of the corresponding nearby contribution point is linearly interpolated in the distorted image. Optical center is set in the center of the image (256.5, 256.5). Radial distortion coefficient *k*_1_ = −0.3, *k*_2_ = 0.1. The camera internal parameter matrix is set to:

(8) $$\left[\begin{array}{ccc} f_{x} & 0 & c_{x}\\ 0 & f_{y} & c_{y}\\ 0 & 0 & 1 \end{array}\right]=\left[\begin{array}{ccc} 450 & 0 & 256.5\\ 0 & 450 & 256.5\\ 0 & 0 & 1 \end{array}\right]$$

**Figure 9:**
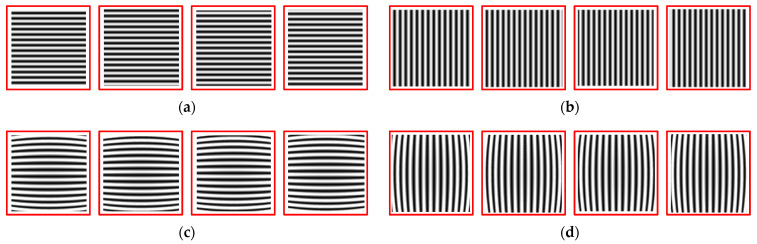

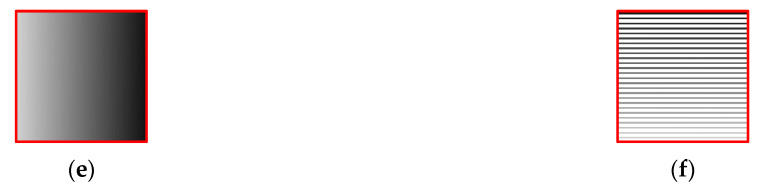
(**a**) Horizontal grating stripes; (**b**) vertical grating stripes; (**c**) horizontal grating fringes obtained by simulating lens distortion; (**d**) vertical grating fringes obtained by simulating lens distortion; (**e**) unwrap the phase of the horizontal fringe to obtain a phase map; (**f**) unwrap the phase of the vertical fringe to obtain a phase map.

**Figure 10:**
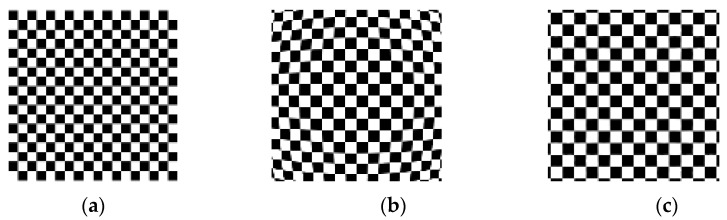
(**a**) checkerboard image; (**b**) checkerboard image obtained by simulating lens distortion; (**c**) refer to the isophase search method and resampling rules to obtain the corrected image.

Obtain horizontal and vertical fringe patterns captured by analog cameras, as well as checkerboard images. Then, unwrap the phase to obtain the corresponding phase map, as shown in Figure 9. Using the same distortion coefficient and camera internal parameters to distort the checkerboard image, refer to the isophase search method and resampling rules to obtain the corrected image, as shown in Figure 10. The simulation results show that it is feasible to use the method in this paper to correct the distorted image when ignoring the sampling error of the camera sensor.

## 3. Experimental Results

### 3.1. Experiments

Set up a system to test the proposed method. The system consists of an LCD monitor (resolution: 1920 pixels × 1080 pixels, pixel size: 0.23 mm × 0.23 mm), camera (resolution: 2256 pixels × 2256 pixels) and a computer. The LCD is located in front of the camera, using auxiliary tools to adjust the LCD and camera positions until they are parallel. At this time, the reason for tangential distortion can be considered as the lens is not completely parallel to the camera sensor.

**Figure 11:**
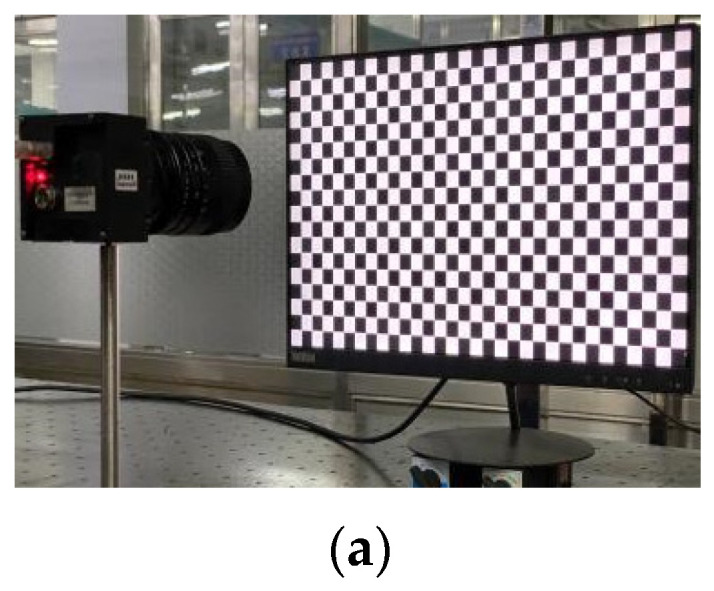

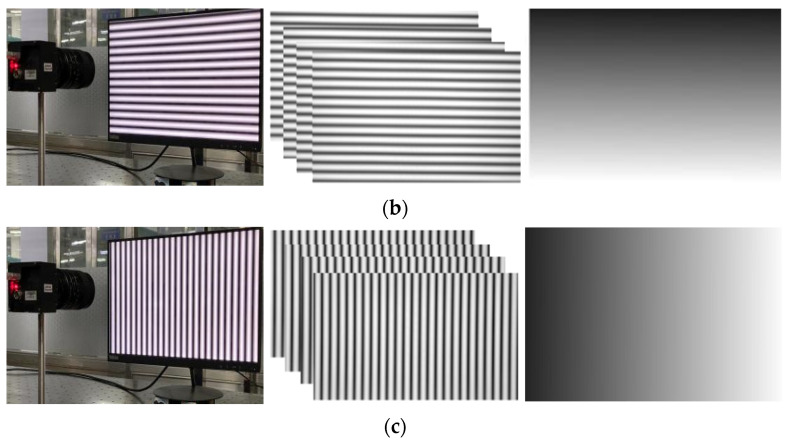
(**a**) Schematic diagram of experimental device; (**b**) the camera captures the horizontal stripes of the LCD display and phase map; (**c**) the camera captures the vertical stripes of the LCD display and phase map.

The sinusoidal grating is displayed on the LCD monitor as a calibration target. Capture four horizontal and vertical fringes. A four-step phase shift algorithm is used to unwrap the phase, as shown in Figure 11. Follow the method in Section 2.3 to smooth the obtained original parameters, and obtain the final correction parameters.

**Figure 12:**
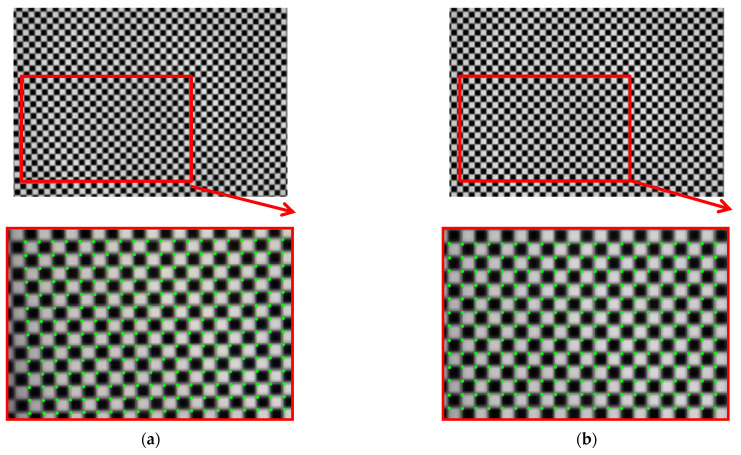
(**a**) Distorted image and its partial enlargement; (**b**) corrected image and its partial enlargement.

According to the phase value of the standard pixels array, the matching pixel is searched in the phase map of the image, and the position coordinates are stored as the original correction parameters. Smooth the coordinate data, and then resample the captured pictures. Revert to the original size. The result is shown in Figure 12. The size of the original image is 2256 × 1556, and the size of the partially enlarged image is 1128 × 778. It can be seen that the corner points in the distorted image have been well corrected.

### 3.2. Comparative Tests

Set up a system to test the proposed method. The system consists of an LCD monitor (resolution: 1920 × 1080, pixel size: 0.23 mm × 0.23 mm), camera (resolution: 2256 × 2256) and a computer. The LCD is located in front of the camera, using auxiliary tools to adjust the LCD and camera positions until they are parallel. At this time, the reason for tangential distortion can be considered as the lens is not completely parallel to the camera sensor. Zhang’s method is a classic method to eliminate image distortion. In order to verify the convenience and accuracy of the method proposed in this paper, the same LCD and checkerboard pattern are used as the calibration target, and the two methods are tested. The method of quantitative evaluation of the correction effect is also the same. The comparison results are shown in Table 1, showing that the method proposed in this paper achieves calibration with an accuracy of 0.39 pixels by only acquiring 8 images. The experimental results show that the method can strike a balance between accuracy and efficiency.

### 3.3. The Impact of Parallelism and Distance on Accuracy

We found that the non-parallelism between the camera and the LCD display is in any direction, and this non-parallelism can be reduced by existing methods. Of course, this parallelism cannot reach 100%. Under normal circumstances, with the help of auxiliary tools (such as high-precision guide rails, micrometers), the LCD display can be placed in an almost parallel position with the camera. The parallelism test is to place the display and the camera at a larger tilt angle. At this time, due to perspective, the phase encoding will no longer be uniform, and the phase will no longer change uniformly. That is, the phase change will be more intense or slow. The standard pixel array of the method in this paper is based on the theory of “uniform phase change”. Therefore, a larger tilt angle will affect the calibration result. This section will discuss the impact of inaccurate parallelism on the accuracy of distortion compensation.

**Figure 13:**
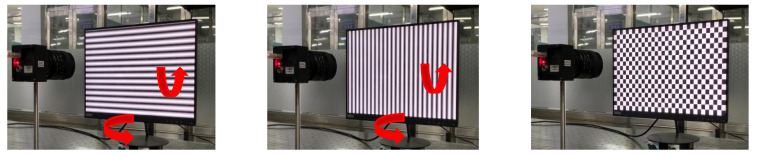
The impact of the parallelism between the camera and the LCD display on the calibration accuracy.

On the basis of the distortion correction experiment, the display of the optical platform (with the aid of a rotating platform) is artificially rotated to a certain angle. It should be noted that this angle is expected to be acceptable, and the pitch and horizontal angles are both controlled within 5° because a large angle will cause serious distortion of the phase encoding. It should also be noted that when shooting a checkerboard image for evaluation, the monitor will be adjusted to the “parallel with the camera” state, as shown in Figure 13.

The test result shows that the evaluation result of corner detection is 0.83 pixels. The error is less than 1 pixel.

The distance between the camera and the display directly affects the density of the stripes encoded on the camera sensor. When the camera’s grayscale resolution is low, it will indeed affect the encoding accuracy. The influence of the distance between the camera and the LCD display on the calibration accuracy can be tested using the existing settings. The experimental results are shown in Table 2.

## 4. Discussion

A fast lens distortion correction method is proposed. The correction is based on the resampling of pixel coordinates. However, traditional evaluation methods, such as reprojection errors, are not applicable. In order to solve the above problems, this paper chooses Zhang’s method based on the distortion model for comparison. The two methods use the same calibration target and the same evaluation method. The correction effect is shown in Figure 12. The method in this paper aims to simplify and stabilize the camera calibration. Usually, the visual measurement task often needs to be calibrated multiple times as required. A simple and stable calibration process can improve efficiency. It can be seen from Table 1 that the traditional method and its improvement method need to take 10 to 20 pictures at different positions, and most of the correction methods based on fringe phase need to construct a distortion model, and it is difficult to obtain high-precision parameters of the model. The method in this paper can realize high-precision lens distortion correction while simplifying the calibration. In the numerical simulation part, the correction result for radial distortion shows the effectiveness of the method. In practical applications, once the device position is fixed, the error can also be eliminated by compensation. This article also proposes an applicable quantitative evaluation method for the model-independent correction method. The correction effect is evaluated through the corner distribution of the checkerboard image.

The checkerboard pattern is displayed on the LCD, so the error introduced by the parallelism is the same as the analysis in the previous proposal. The accuracy of corner extraction will indeed affect the evaluation results. This paper also tried several corner extraction algorithms. According to the results of a literature search, we found that the corner extraction algorithm based on the gradient of image gray value can extract the corner points from the noise with high precision. It can be seen from Figure 5 that the gray level changes around the corner points are uniform and can be extracted according to a unified standard. In the evaluation of several methods in this article, the corner extraction methods used are consistent with Zhang’s camera calibration method. Therefore, we can expect that the method proposed in this article can provide higher accuracy under the same test conditions.

In addition, we have analyzed or verified the accuracy factors that may affect the calibration and evaluation. For example: the parallelism of the camera and the LCD, the distance between the camera and the LCD, the nonlinearity of the camera response intensity, the accuracy of corner extraction, etc. The results show that the method for solving the problem is robust and can still control the accuracy error to less than 1 pixel.

## 5. Conclusions

In this paper, a new lens distortion correction method using vertical and horizontal fringe patterns is proposed. The key to the distortion correction is pixel-by-pixel analysis and correction. So, a four-step phase shift algorithm is used to calculate the envelope phase. The characteristic points are detected by the isophase line detection method, and the standard pixels array is constructed by the interpolation method. Finally, resampling is performed pixel by pixel. The experimental results show that this method provides higher accuracy compared with the traditional method. In addition, taking the corners of the checkerboard as the control points, a quantitative evaluation method of the correction is proposed. The effect is evaluated by constructing strict non-distortion feature points. The evaluation results show that the average correction error is 0.39 pixels. Different from other methods, the parameter acquisition and correction process of the method in this paper are derived from the actual data of each pixel of the sensor and image, rather than a strict mathematical model. The results of experiment and comparison show the effectiveness and superiority of the method.

We considered further related work. First, the proposed method is still applicable to mixed radial and tangential distortions, but the chromatic aberration correction of the lens is also an important measure that affects the image quality. The lens has different refraction to light of different wavelengths, so the chromatic aberration of the lens can be corrected by using the characteristics of the LCD pixels.

## Figures and Tables

**Table 1:** Comparisons.

Method	Results (Partial Enlargement)	RMS	Time Consuming	Description
Zhang	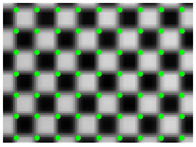	1.28 pixels	1.1 s	The accuracy is related to the quality and position of the input calibration images.
Tsai	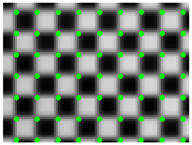	1.83 pixels	2.9 s	It is not possible to calibrate all external parameters through a plane, and non-linear calculations may make the results unstable. Only consider radial distortion.
Thirthala	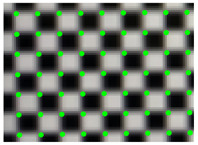	3.81 pixels	1.9 s	The position of the distortion center affects the correction result.
The proposed method	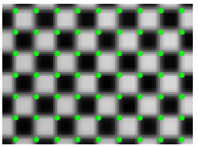	**0.39 pixels**	2.2 s	The position of the calibration targets is fixed. Simple operation and high precision.

**Table 2:** The influence of the distance between the camera and the LCD.

**Distance**	30 cm	35 cm	40 cm	45 cm	50 cm
**RMS**	0.40 pixels	0.39 pixels	0.44 pixels	0.47 pixels	0.55 pixels

## Data Availability

The study did not report any data.
